# TALE gene family: identification, evolutionary and expression analysis under various exogenous hormones and waterlogging stress in *Cucumis sativus* L.

**DOI:** 10.1186/s12870-024-05274-3

**Published:** 2024-06-15

**Authors:** Sheraz Ahmad, Khushboo Khan, Ibrahim A. Saleh, Mohammad K. Okla, Ibrahim A. Alaraidh, Hamada AbdElgawad, Muhammad Naeem, Naveed Ahmad, Shah Fahad

**Affiliations:** 1 College of Plant Protection, Yangzhou University, 48 Wenhui East Road, Yangzhou, Jiangsu 225009 PR China; 2 Faculty of Crop Production Sciences, The University of Agriculture Peshawar, Peshawar, Khyber Pakhtunkhwa 25120 Pakistan; 3 Faculty of Science, Zarqa University, Zarqa, 13110 Jordan; 4 Botany and Microbiology Department, College of Science, King Saud University, P.O. Box 2455, Riyadh, 11451 Saudi Arabia; 5 Integrated Molecular Plant Physiology Research, Department of Biology, University of Antwerp, Antwerp, 2020 Belgium; 6 Department of Plant Science, School of Agriculture and Biology, Shanghai Jiao Tong University, Shanghai, 200240 China; 7 Joint Center for Single Cell Biology, Shanghai Collaborative Innovation Center of Agri-Seeds, School of Agriculture and Biology, Shanghai Jiao Tong University, 800 Dongchuan Road, Shanghai, 200240 China; 8 Department of Agronomy, Abdul Wali Khan University Mardan, Mardan, Khyber Pakhtunkhwa 23200 Pakistan

**Keywords:** Cucumber, TALE, Waterlogging, Hormones, Bioinformatics

## Abstract

**Background:**

Three Amino acid Loop Extension (TALE) belongs to the homeobox group of genes that are important constituents of plant systems. The TALE gene family is instrumental not only in growth and development but also plays an essential role in regulating plant response to environmental adversaries.

**Results:**

In the present study, we isolated 21 *CsTALE* genes from the cucumber (*Cucumis sativus* L.) genome database. Bioinformatics tools were put in place to understand the structural and functional components of the *CsTALE* gene family. The evolutionary analysis dissected them into seven subclades (KNOX-I, KNOX-II, and BELL-I to BELL-V). The *c*is*-*acting elements in the promoter region of *CsTALE* genes disclosed that they are key regulators of hormonal and stress-related processes. Additionally, the STRING database advocated the concerting role of CsTALE proteins with other key transcription factors potent in plant developmental biology. The CsmiR319 and CsmiR167a-3p targeting the *CsTALE15* and *CsTALE16*, respectively, further assert the importance of the *CsTALE* gene family posttranscriptional-related processes. Tissue-specific gene expression unfolded the fundamental involvement of *CsTALE* genes as they were expressed throughout the developmental stages. Under waterlogging stress, the *CsTALE17* expressed significantly higher values in WL, WL-NAA, and WL-ETH but not in WL-MeJA-treated samples.

**Conclusions:**

The present study reveals the evolution and functions of the *CsTALE* gene family in cucumber. Our work will provide a platform that will help future researchers address the issue of waterlogging stress in the Yangtze River Delta.

**Supplementary Information:**

The online version contains supplementary material available at 10.1186/s12870-024-05274-3.

## Introduction

TALE, a class of transcription factors, is found in eukaryotes fine-tuning key regulatory processes [1]. An exceptionally conserved sequence, later dubbed the homeobox, was identified by Gehring et al. (1987) [2] by other researchers as a result of gene mutations leading to a similar phenotype. The family gene contains KNOX and BLH/BELL domain-encoded proteins [3], consisting of physically and functionally similar heterodimers. TALE family genes encode 63-amino-acid homeobox domains. This family is known as the homeobox protein superfamily because of the three-amino acid loop extension that connects the homeodomain’s first and second helices [4]. The *Arabidopsis thaliana* (AtTALE) transcription factors are classified into two subfamilies: the BELL and the KNOX [4]. Specific interactions between these two subfamilies govern downstream genes for various biological roles [5]. The four domains that make up the KNOX subfamily—KNOX1, KNOX2, ELK, and Homeobox KN—are found in plants [6].

The initial homeobox in plants was identified as Kn-1 (Knotted-1) by [7]. SAM production was shown to be significantly inhibited by a mutation in the STM (*SHOOTMERISTEMLESS*) [3]. KNAT7 played a role in secondary cell wall biosynthesis [8, 9]. Cotton fiber synthesis is controlled by GhKNL1, a member of the TALE family [10]. Flowers, xylem differentiation, and hormone effects may be regulated by Arabidopsis KNOX Class I family genes (STM, KNAT2, BREVIPEDICELLUS (BP)/ KNAT1, and KNAT6) [10]. The roots, stems, leaves, and flowers are the locations where the Class II KNOX genes are expressed among the angiosperms. This set of genes’ primary function is to control the organ differentiation process in plants [3, 10]. Gibberellic acid (GA), cytokinin (CK), and lignin are likewise regulated by KNOX proteins [11]. Floral development and metamorphosis are regulated in significant ways by members of the BELL family. Different from one another, overexpression of the BLH3 gene causes flowers to open earlier, and overexpression of the BLH6 gene causes delayed flowering [12]. Selectively combining KNOX and BELL forms heterodimer heteromers that bind to target sequences. Various pairings of two subfamilies can control distinct sets of genes to carry out their roles in plants [5]. A key enzyme in the GA biochemical pathway, ga20ox1, was inhibited in its expression by the BELL/KNOX dimer StBEL5 / POTH1 [3]. ATH1 and PENNYWISE (PNY), both BELL family members, regulated plant meristem development similar to STM [8]. *A. thaliana* BLH1 and KNAT3 proteins combine to produce heterodimers that impact seed germination and development as well as the expression level of genes responsive to ABA [13, 14]. The development of pistil marginal tissue is regulated by KNAT1 through interactions with RPL, FUL, and AP [10].

*C. sativus* is the third most popular vegetable crop, growing on 1.98 million hectares and producing 93 million tons. China accounts for 78% of the world’s production, with 72 million tons out of a total of 93 million tons [15, 16]. Flooding is the biggest threat to agriculture and food security worldwide. Flood losses in low- and lower-middle-income nations were USD 21 billion between 2008 and 2018, 19% of all agricultural losses [17–19]. Ethylene pretreatments increased hypoxia tolerance, rosette diameters, and root tip re-grow after recovery in *A. thaliana* [20–22]. Other hormones, such as auxin and methyl jasmonate, were also reported to regulate plant response to waterlogging stress [21, 23–26]. Thus far, not a great deal of literature is available on the involvement of the TALE gene family in parthenocarpy and waterlogging.

In this work, we have conducted a detailed analysis of the *CsTALE* gene family using numerous bioinformatic tools. Applying phytohormones (IAA, CK, and GA) induces parthenocarpy in cucumber. We have analyzed the expression of *CsTALE* genes in response to IAA, CK, and GA. Additionally, the expression of *CsTALE* genes was analyzed in different tissues and under waterlogging stress. Our conducted study will be of great interest to future research work, particularly in waterlogging.

## Results

### Identification of *TALE* genes in the cucumber genome

To isolate the TALE proteins from the cucumber genome database (CGD), we utilized the BLASTP program. The retrieved proteins were further investigated for the Homeobox_KNOX (HB_KN) domain. Proteins lacking the HB_KN domain were discarded, and following that, we were left with 21 CsTALE members. The chromosomal position was investigated in the CGD. The ExPASy online server was used for the number of amino acids, molecular weight, and isoelectric point. Finally, the Subcellular analysis was performed, and we observed that all the CsTALE proteins reside in the nucleus (Table 1).

**Table 1:** Physiochemical properties of CsTALE proteins

Locus ID	Gene	Chr.	Start	End	AA	MW (kDa)	PI	SL
Csa1G004870	*CsTALE1*	1	839,762	845,531	314	35207.52	5.38	Nucleus
Csa1G118890	*CsTALE2*	1	9,012,090	9,017,334	820	91560.96	6.11	Nucleus
Csa2G034560	*CsTALE3*	2	3,335,281	3,338,884	327	37369.31	5.04	Nucleus
Csa2G249860	*CsTALE4*	2	12,228,795	12,231,645	687	75512.65	5.98	Nucleus
Csa2G287110	*CsTALE5*	2	13,696,177	13,702,147	661	72975.29	5.79	Nucleus
Csa2G348930	*CsTALE6*	2	15,770,430	15,774,065	169	19521.10	7.09	Nucleus
Csa2G351760	*CsTALE7*	2	16,208,915	16,210,622	492	55228.72	5.76	Nucleus
Csa2G352430	*CsTALE8*	2	16,306,379	16,310,046	406	46520.65	6.11	Nucleus
Csa3G733340	*CsTALE9*	3	27,883,158	27,886,801	486	54935.44	6.81	Nucleus
Csa3G782620	*CsTALE10*	3	30,260,873	30,264,668	722	79328.86	6.66	Nucleus
Csa4G297540	*CsTALE11*	4	12,242,105	12,246,510	467	53319.24	6.62	Nucleus
Csa4G645830	*CsTALE12*	4	21,808,266	21,812,207	375	43152.31	5.91	Nucleus
Csa4G652730	*CsTALE13*	4	22,778,556	22,782,578	476	52996.85	5.98	Nucleus
Csa5G152860	*CsTALE14*	5	4,854,760	4,858,291	612	67568.45	5.98	Nucleus
Csa5G156150	*CsTALE15*	5	5,425,321	5,428,901	307	34445.81	6.06	Nucleus
Csa5G600390	*CsTALE16*	5	21,925,448	21,932,133	330	37407.15	5.01	Nucleus
Csa6G426360	*CsTALE17*	6	20,030,373	20,034,057	704	76970.39	6.97	Nucleus
Csa6G487770	*CsTALE18*	6	22,977,392	22,982,614	702	76464.81	6.46	Nucleus
Csa6G513430	*CsTALE19*	6	26,530,396	26,533,379	357	39895.86	5.55	Nucleus
Csa7G041370	*CsTALE20*	7	2,231,073	2,235,700	356	40080.13	6.44	Nucleus
Csa7G432650	*CsTALE21*	7	17,316,741	17,319,618	554	61892.46	5.64	Nucleus

Chromosome: Chr., Amino acid: AA, Molecular Weight: MW, Isoelectric point: PI, Subcellular location: SL

### *CsTALE* Gene family conserved domain analysis

**Fig. 1:**
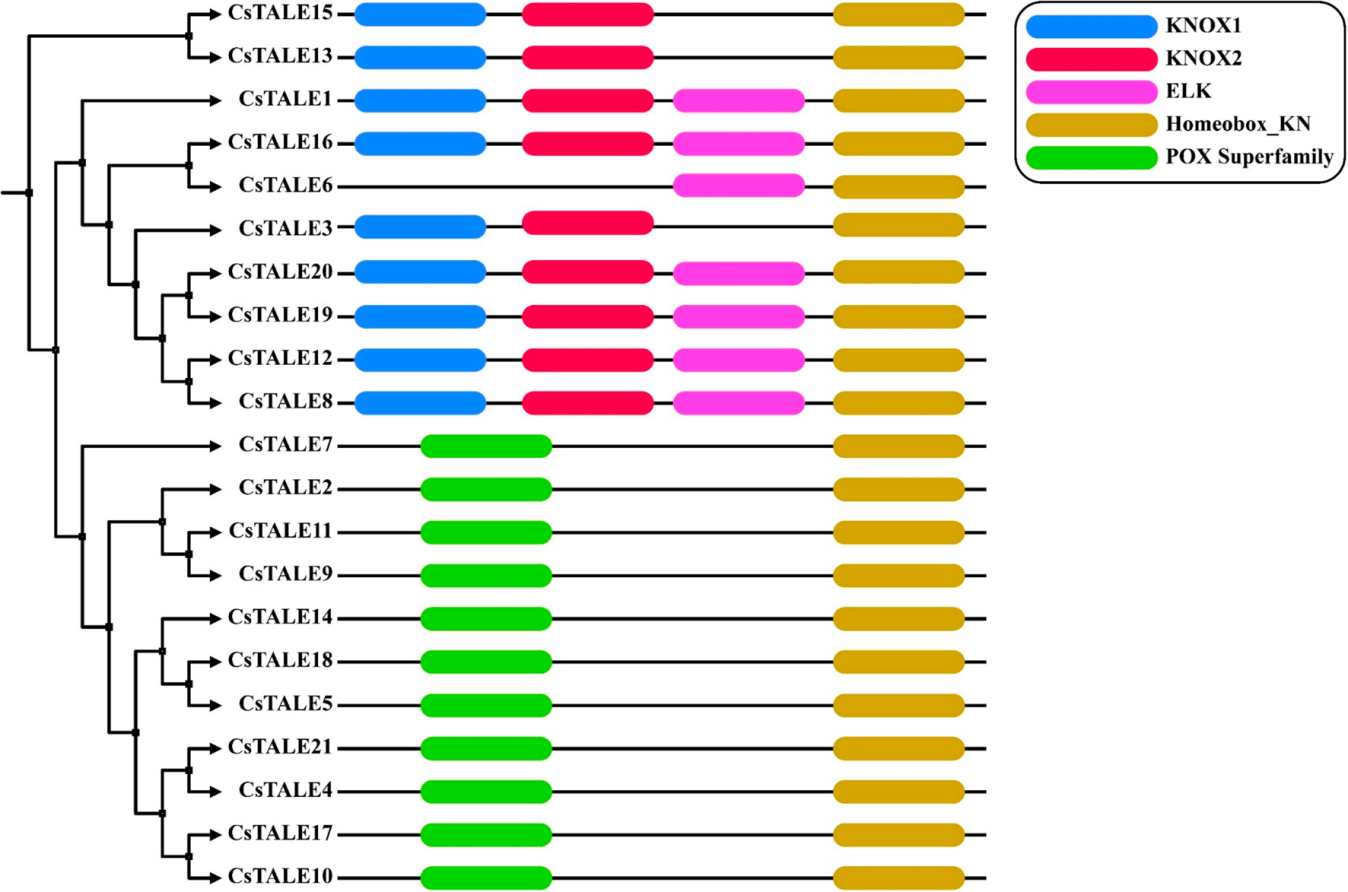
Schematic representation of the conserved Domains of *CsTALE* genes in *C. sativus*. The conserved domains were identified using the NCBI conserved domain search

In order to gain a deep insight into the diversity of *CsTALE* gene functions, we used the MEME online server (http://meme-suite.org/tools/meme*)* to predict conserved motifs in CsTALE proteins. A total of five different motifs were found in the CsTALE proteins. Four members of the *CsTALE* gene family (*CsTALE1*, *CsTALE8, CsTALE12, CsTALE16, CsTALE19*, *CsTALE20*) were found with the highest number of KNOX1, KNOX2, ELK, Homeobox_KN and POX Superfamily domains. Furthermore, only CsTALE proteins such as CsTALE3, CsTALE13, and CsTALE15 possess KNOX1, KNOX2, and Homeobox_KN domains, and the CsTALE6 with ELK and Homeobox_KN domains. Finally, the highest number of CsTALE proteins was found with the Homeobox_KN and POX Superfamily domains (Fig. 1); the *A. thaliana* conserved domains of the two subfamilies (KNOX, BELL) are attached in the supplementary data Figure S1.

### Evolutionary analysis of *CsTALE* genes

**Fig. 2:**
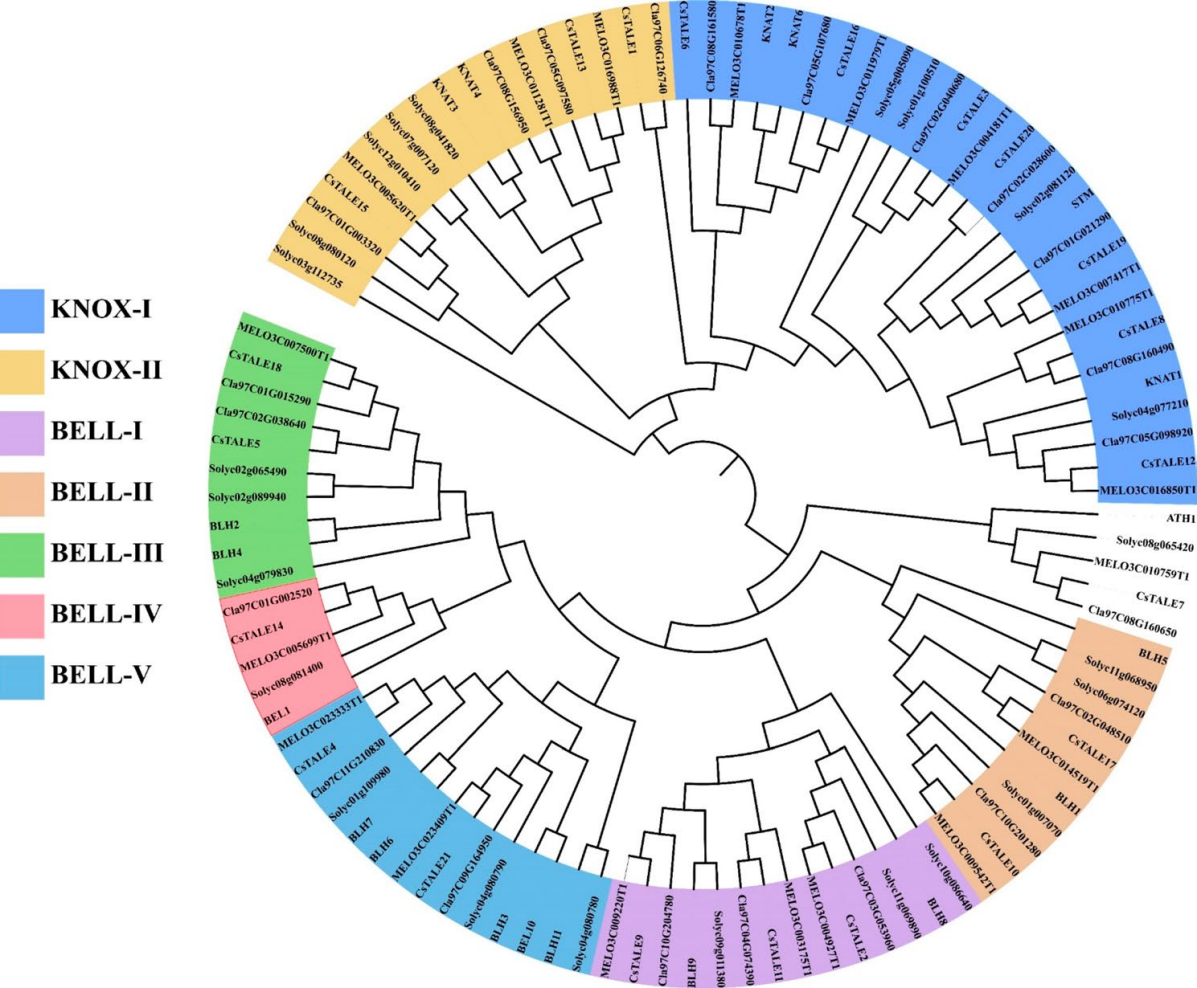
Phylogenetic analysis of the *CsTALE* genes family. The phylogenetic tree was generated using the amino-acid sequences via ML methods. All *CsTALE* were classified into seven groups, and the final tree was displayed using ITOL.

The phylogeny analysis was conducted to examine the evolutionary characteristics of CsTALE with other species, including *A. thaliana, Cucumis melo, Citrullus lanatus*, and *Solanum lycopersicum.* The highest number of *TALE* genes were attributed to KNOX-1, followed by KNOX-II (Fig. 2). The BELL group of subfamilies consists of 5 additional subfamilies (BELL-I to BELL-V). The BELL-I and BELL-V contain the highest number of TALE genes, whereas the BELL-II, BELL-III, and BELL IV exhibit a decent amount of TALE genes

### Synteny analysis among *CsTALE* genes

**Fig. 3:**
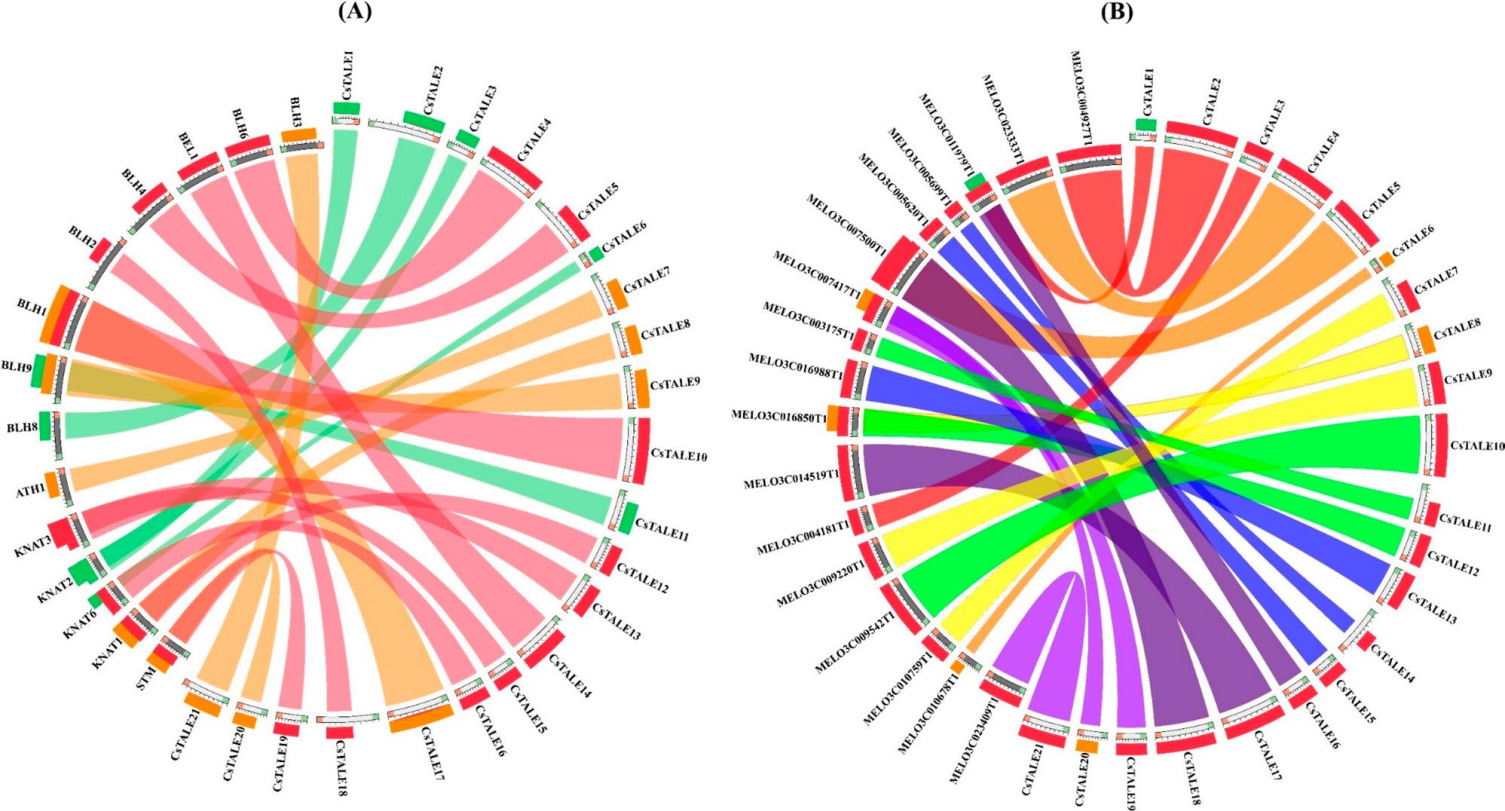
Synteny analysis of TALE genes between **(A) ***C. sativus* and *A. thaliana* and **(B) ***C. sativus* and *C. melo*. The chromosomes of *C. sativus*, *A. thaliana*, and *C. melo* are arranged as a circle. Colored lines represent syntenic occurrences of TALE genes

Synteny links between the cucumber, melon, and Arabidopsis genomes were also examined to determine the likely roles of the *CsTALE* genes. In both the Arabidopsis (~ 50%) and cucumber (~ 86%) genomes, all of the *CsTALE* genes had synteny links, as shown in (Fig. 3A). Similarly, high syntenic links between cucumber (~ 70%) and melon (~ 55%) were also observed (Fig. 3B). During genome evolution, these wide synteny relations at the gene level can demonstrate the close evolutionary links and extensive rearrangement events of the cucumber chromosomes.

### Chromosomal localization of *CsTALE* genes

**Fig. 4:**
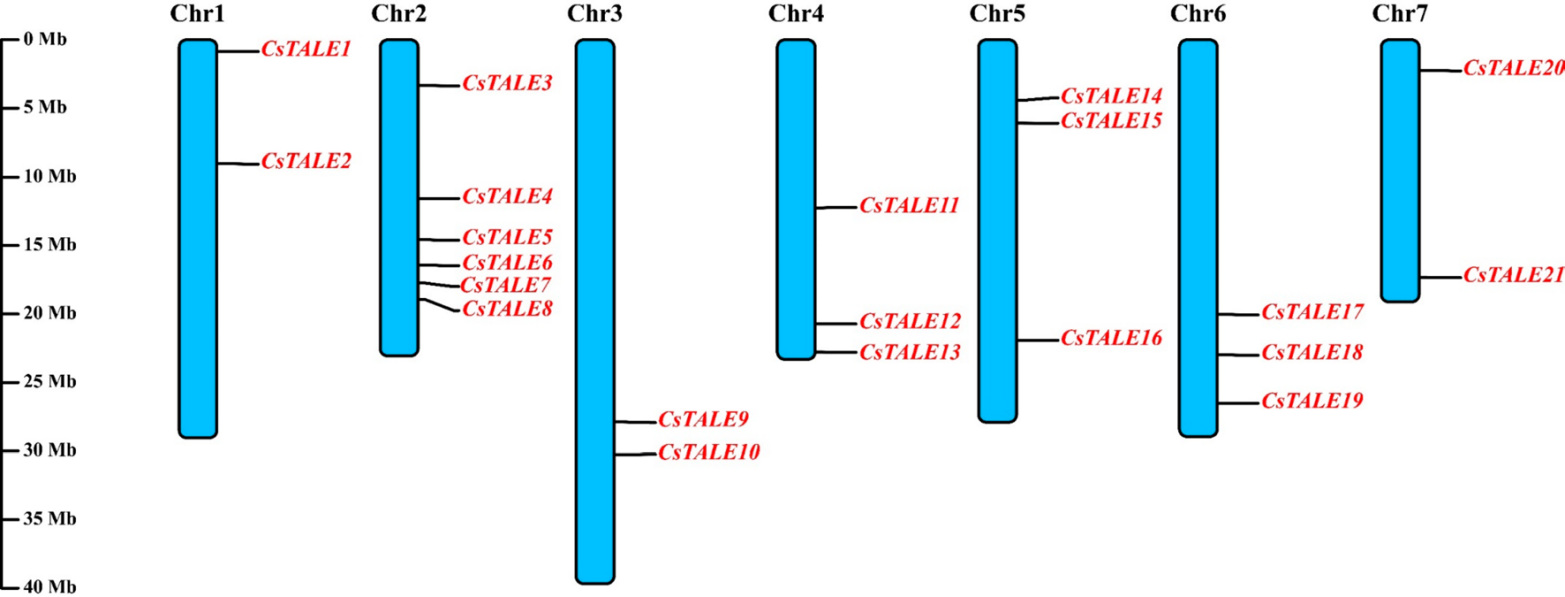
Localization of *CsTALE* genes on chromosomes. There are 7 chromosomes in cucumber, and the number of chromosomes is indicated at the top of each chromosome. The relative positions of *CsTALE* genes are marked on the chromosomes. The schematic representation was made using TBtools-II (Version 1.098765)

Chromosomal localization is performed to visualize the physical location of *CsTALE* genes. The cucumber genome contains 7 chromosomes, and the *CsTALE* genes were distributed in random positions (Fig. 4). Chromosome 2 possesses the highest number of genes (six genes), followed by chromosomes 4, 5, and 6 (3 genes each). Chromosomes 1, 3, and 7 only accounted for 2 *CsTALE* genes each.

### Gene structure and motif analysis

**Fig. 5:**
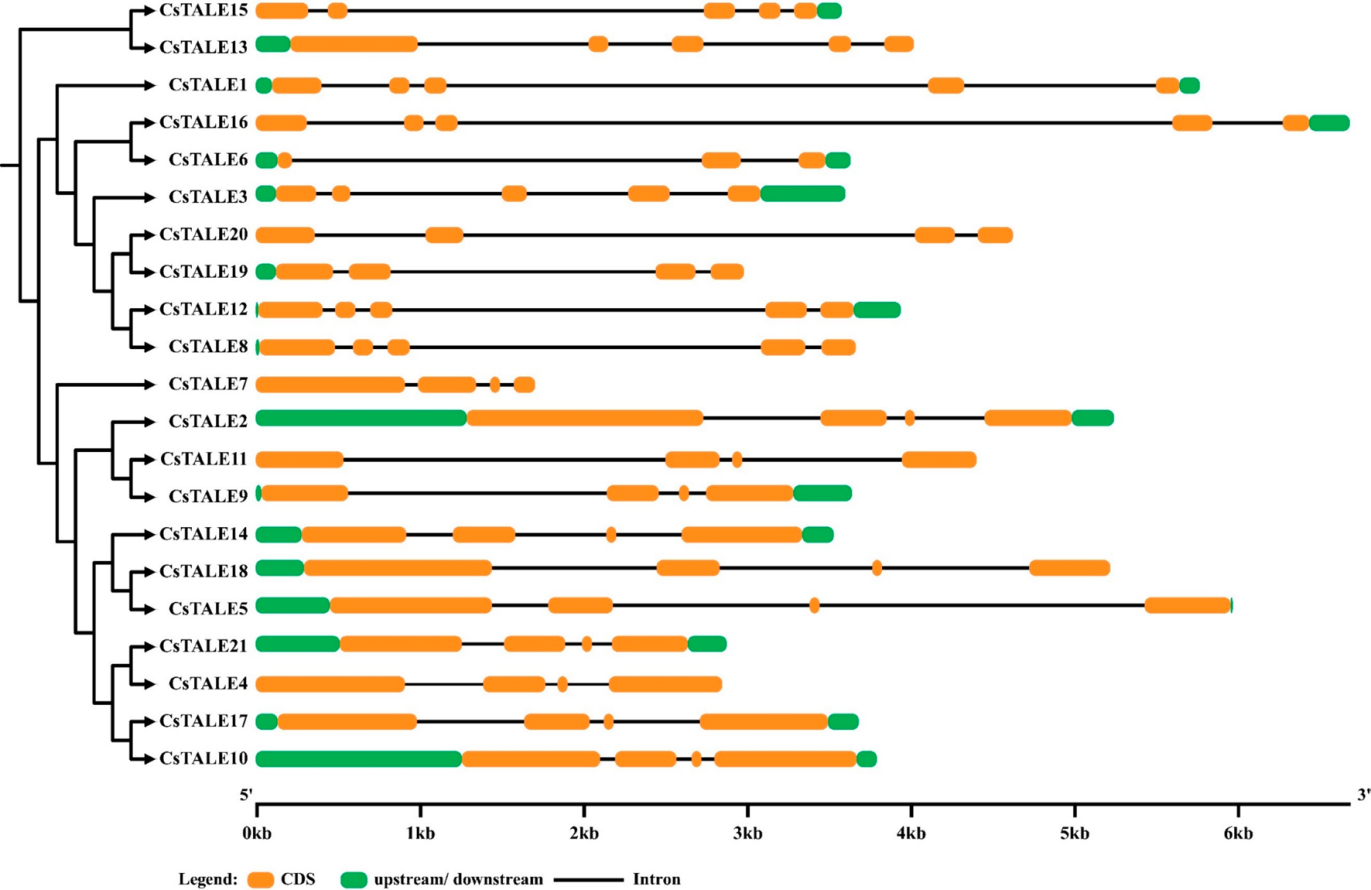
Schematic representation of gene structure. The gene structure analysis was obtained from the gene display structure

The gene structure analysis is vital to understanding the evolutionary composition of a certain gene or family. The *CsTALE* gene’s structure was drawn using genomic and CDS sequences. The exons (CDS) and introns were randomly distributed as disclosed by structural analysis (Fig. 5). For instance, the majority of the *CsTALE* genes possess 4–5 exons in their structure. On the other hand, 2–3 introns were recorded in the bulk of the *CsTALE* genes.

**Fig. 6:**
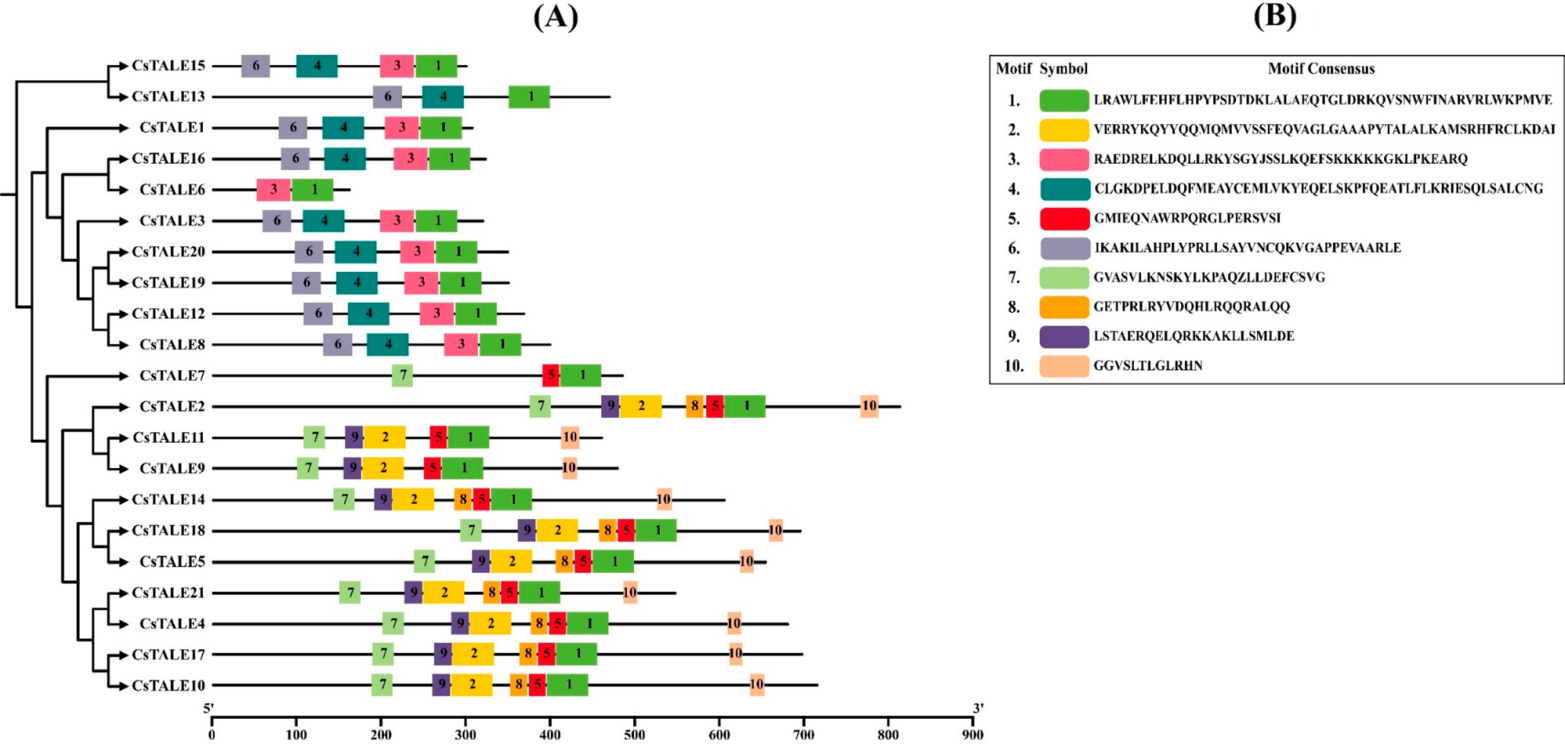
Schematic representation of motifs distribution in *CsTALE* gene family. The distribution of the motifs was obtained from the MEME online server

The MEME online server predicted a total of 4 motifs in the protein sequence of CsTALE (Fig. 6). The highly conserved motif 1 (Homeobox) was presented in all the CsTALE proteins, whereas motif 2 (POX) was found missing in several. The motif 3 (KNOX II) was recorded in predominant numbers, whereas the motif 4 (KNOX I) was absent in many CsTALE proteins.

### Gene ontology analysis of *CsTALE* genes

**Fig. 7:**
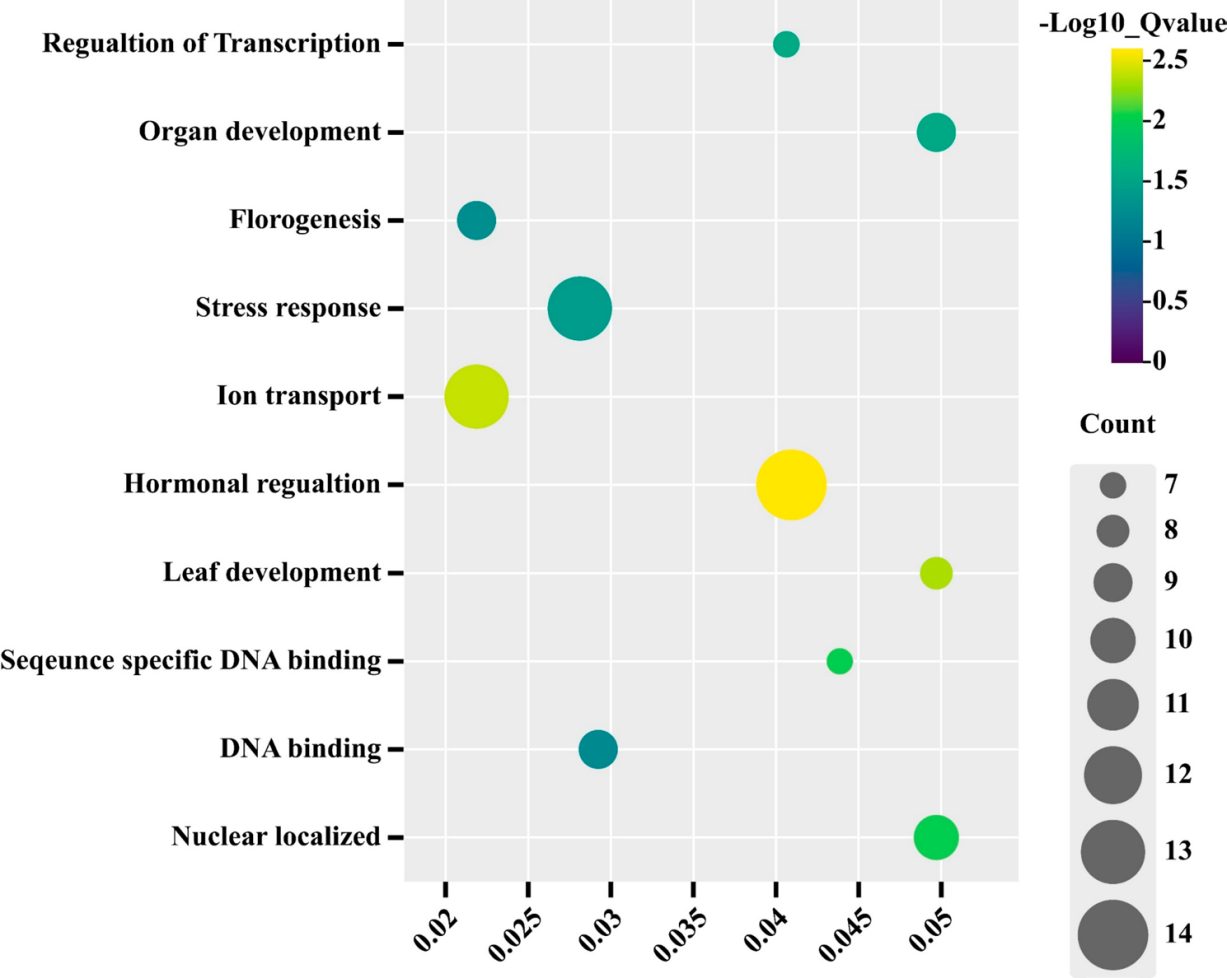
GO enrichment analysis of *CsTALE* genes in cucumber. The data represented biological processes, molecular functions, cellular components, and their localization proportions

The gene ontology (GO) analysis comprised three categories: biological functions, molecular functions, and cellular components. The BF predicted that *CsTALE* genes are, by and large, involved in flowering, hormonal regulation, organ development, and stress response (Fig. 7). The *CsTALE* genes exhibit DNA binding ability as suggested by the molecular function. According to CC, all the *CsTALE* genes reside in the nucleus.

### Promoter analysis of *CsTALE* genes

**Fig. 8:**
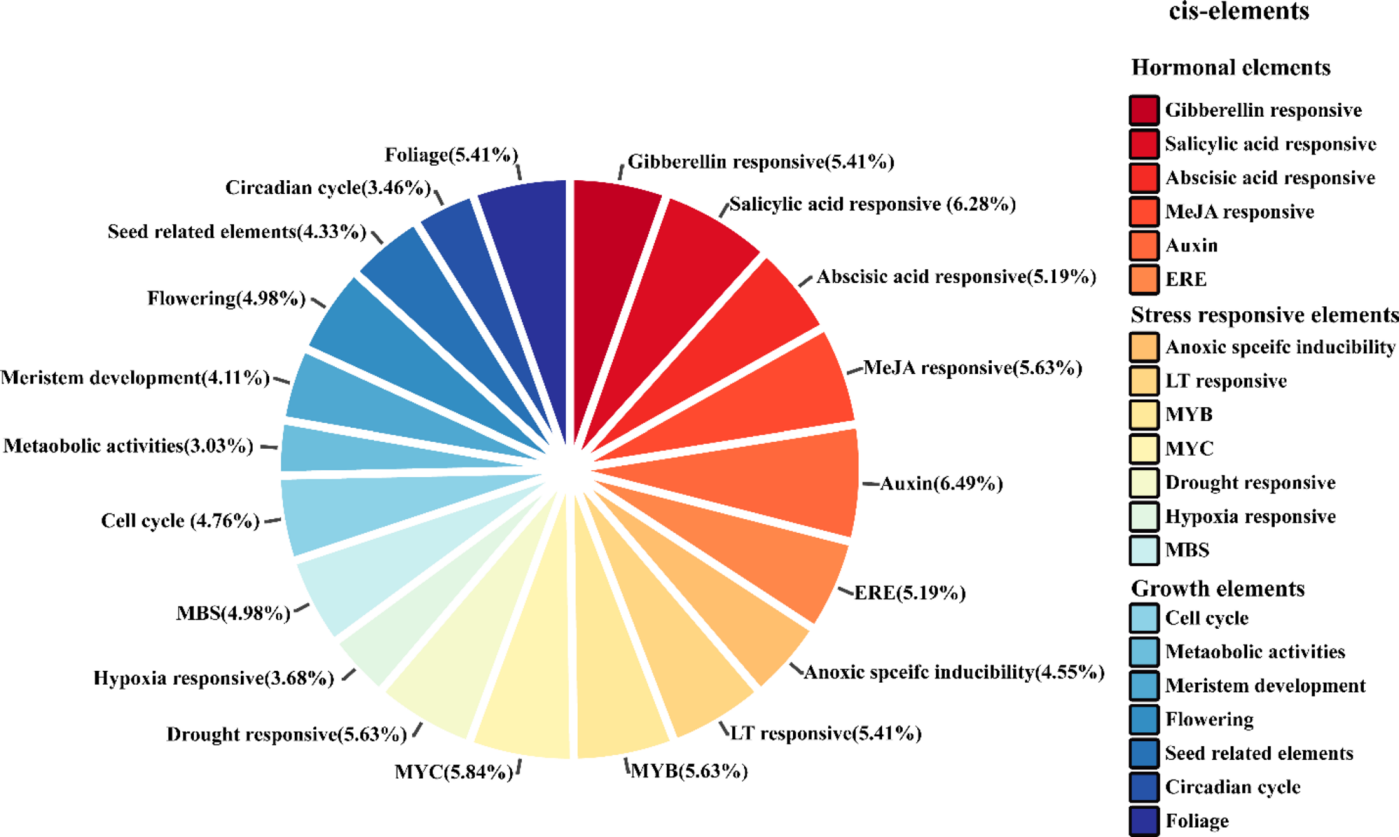
The *cis-acting* elements of *CsTALE* genes in cucumber. The data represented hormonal regulation, stress-responsive, and growth-related elements

The 1.5 kb promoter region upstream of *CsTALE* genes was retrieved from the cucumber genome database, and the predicted *cis-*elements were searched by the PlantCARE database. The predicted *cis-*acting elements were categorized into different groups (hormonal, stress, and growth). The hormonal-related elements contain *cis-*elements for almost all the major hormones, including GA, SA, ABA, MeJA, auxin, and ERE (ethylene-responsive elements). The *CsTALE* genes could be responsive to a variety of environmental stresses. For instance, anoxia, low temperature, drought, and hypoxia. The drought-responsive element MBS is also presented in the upstream region of the *CsTALE* gene (Fig. 8). Could be of a versatile nature, the *CsTALE* genes tailor key growth processes such as cell cycle, metabolic activities, flowering, circadian cycle, and foliage formation.

### Interactive protein network analysis

**Fig. 9:**
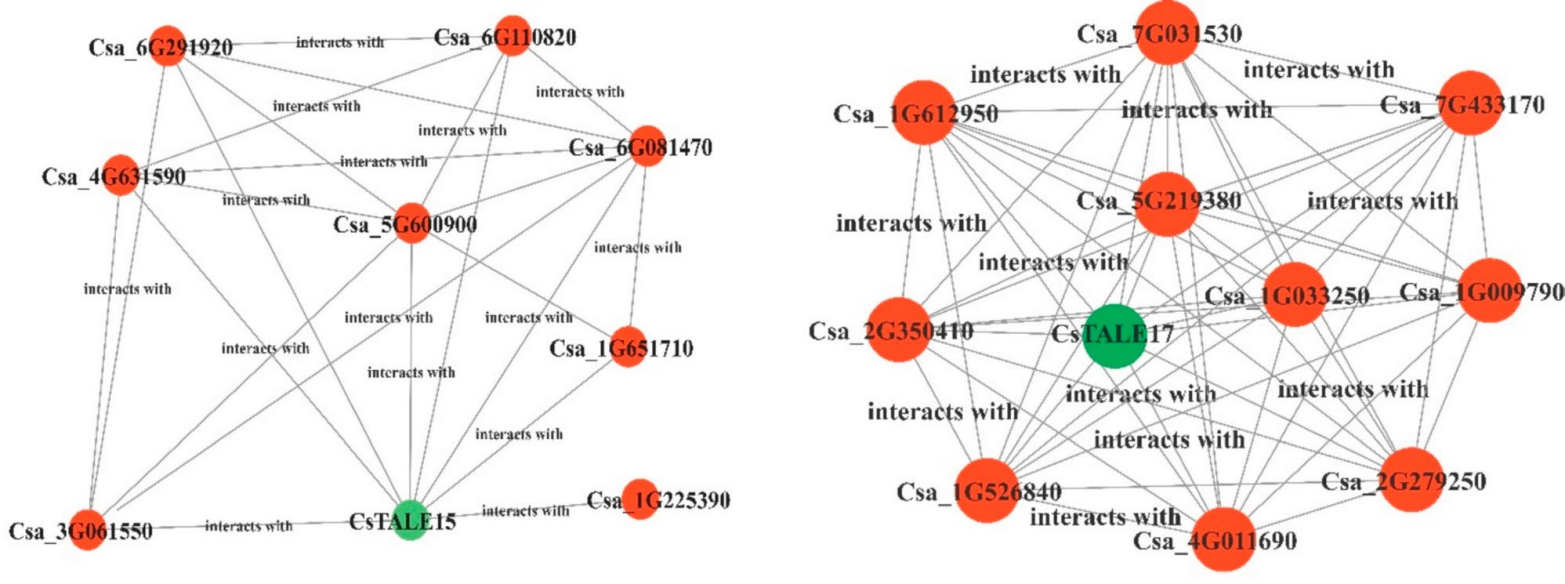
The predictive functional partners of CsTALE-proteins. The CsTALE15 and CsTALE17 were used as a reference in the String online tool for functional predictor analysis

The interactive protein analysis via the STRING online database is a key tool for identifying the functional partners. Herein, we referred to two CsTALE proteins (CsTALE15 and CsTALE17) to recognize their functional predictors (Fig. 9). For CsTALE15, an array of key proteins was found in the interaction. Amongst them, Csa1G225390 (Receptor-like protein kinase), Csa3G061550 (Scarecrow-like 1 transcription factor), Csa1G651710 (Flowering locus T-like 2) and Csa5G600900 (KELCH DOMAIN-CONTAINING F-BOX PROTEIN). The CsTALE17 was also found in interaction with several key transcription factors. The Csa2G350410 (Trihelix transcription factor GT-1), Csa1G526840 (UDP-glycosyltransferase 1), Csa7G031530 (CONSTANS-like 9), and Csa1G033250 (Dof zinc finger protein).

### 10 targeted miRNA of *CsTALE* genes

**Fig. 10:**
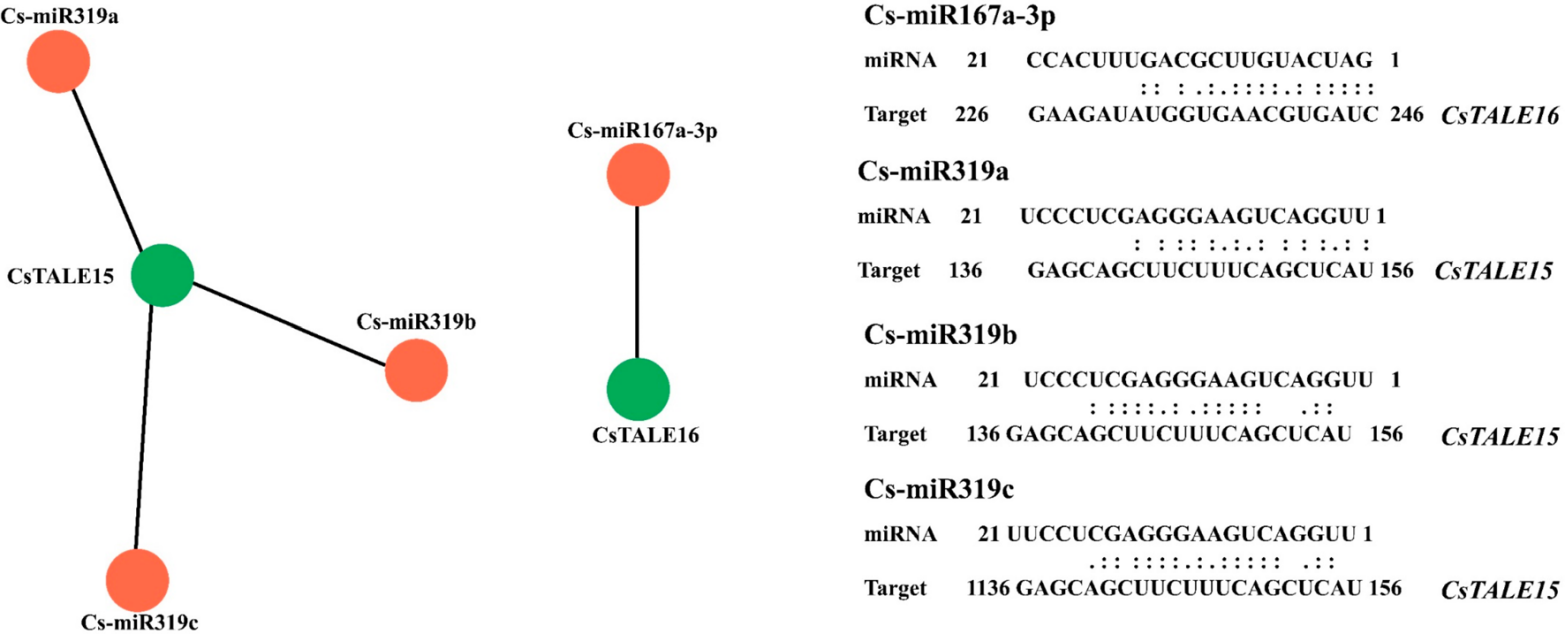
Schematic representation of (A) miRNA network interaction with *CsTALE* genes. The miRNA network was presented through Cytoscape (version 3.9.1)

The miRNA prediction is vital to understand the posttranscriptional regulatory mechanism of *CsTALE* genes. We used the CDS sequences of *CsTALE* genes to identify their sponged miRNAs. The analysis revealed that the miRNA319 family (Cs-miR319a, CsmiR319b, and Cs-miR319c) targeted the *CsTALE15* gene (Fig. 10). Additionally, the Cs-miR167a-3p targets the *CsTALE16* gene, as can be seen from the outcome of the predictor analysis.

### 11 tissue-specific expression analysis of *CsTALE* genes

**Fig. 11:**
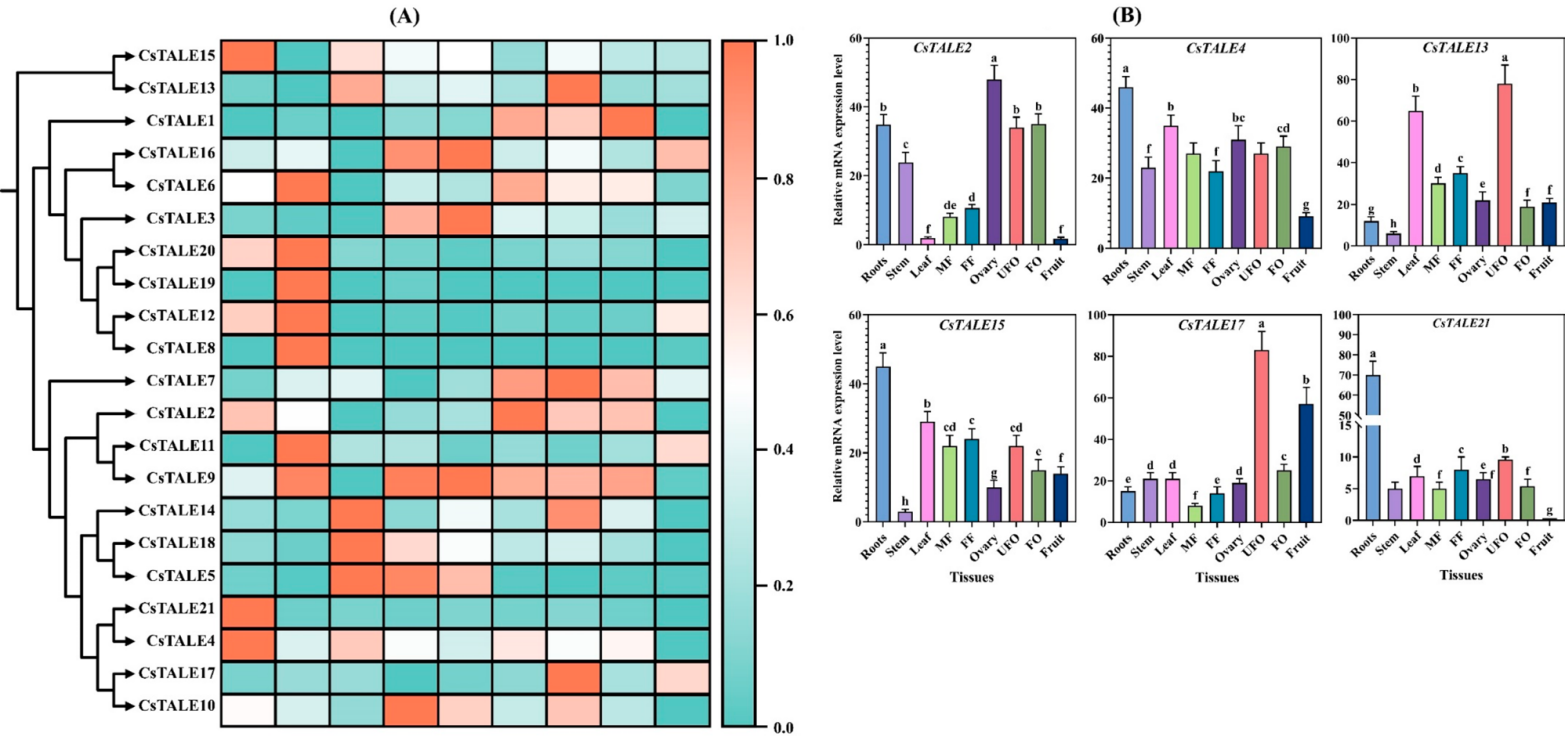
Organs-specific expression analysis of *CsTALE* genes family. (**A**) Heatmap representation of *CsTALE* genes in developmental stages. The log2 transformation method normalized and converted the RPKM values. (**B**) qRT-PCR expression analysis of selected *CsTALE* genes in different tissues

The expression of *CsTALE* genes was investigated in different cucumber tissues to map their general regulatory role in growth. The mRNA abundance data of different tissues was retrieved from the cucumber genome database (Fig. 11A). Varied expression of *CsTALE* genes was observed in different tissues. For instance, in roots, *CsTALE2, CsTALE4, CsTALE12, CsTALE15, CsTALE20* and *CsTALE21* were expressed dominantly. In stem, the *CsTALE6, CsTALE8, CsTALE9, CsTALE11, CsTALE12* and *CsTALE20* displayed high mRNA level. The *CsTALE5, CsTALE14*, and *CsTALE18* were among the highly expressed genes in the leaf. The *CsTALE3, CsTALE5, CsTALE9, CsTALE10*, and *CsTALE16* showed expression abundance in the male flower (MF) and female flower (FF). In the reproductive tissue (ovary, fertilized ovary [FO] and unfertilized ovary [UO]), the *CsTALE1, CsTALE2, CsTALE6, CsTALE7*, and *CsTALE9* were highly expressed. Only *CsTALE17* displayed a high level of expression in fruit tissue (Fig. 11A).

Further, we selected six genes from the heatmap based on their expression to validate the data by qRT-PCR analysis. The qRT-PCR expression analysis revealed an almost similar expression trend to that with the heatmap (Fig. 11B).

### 12 *CsTALE* genes expression under waterlogging stress and hormonal applications

**Fig. 12:**
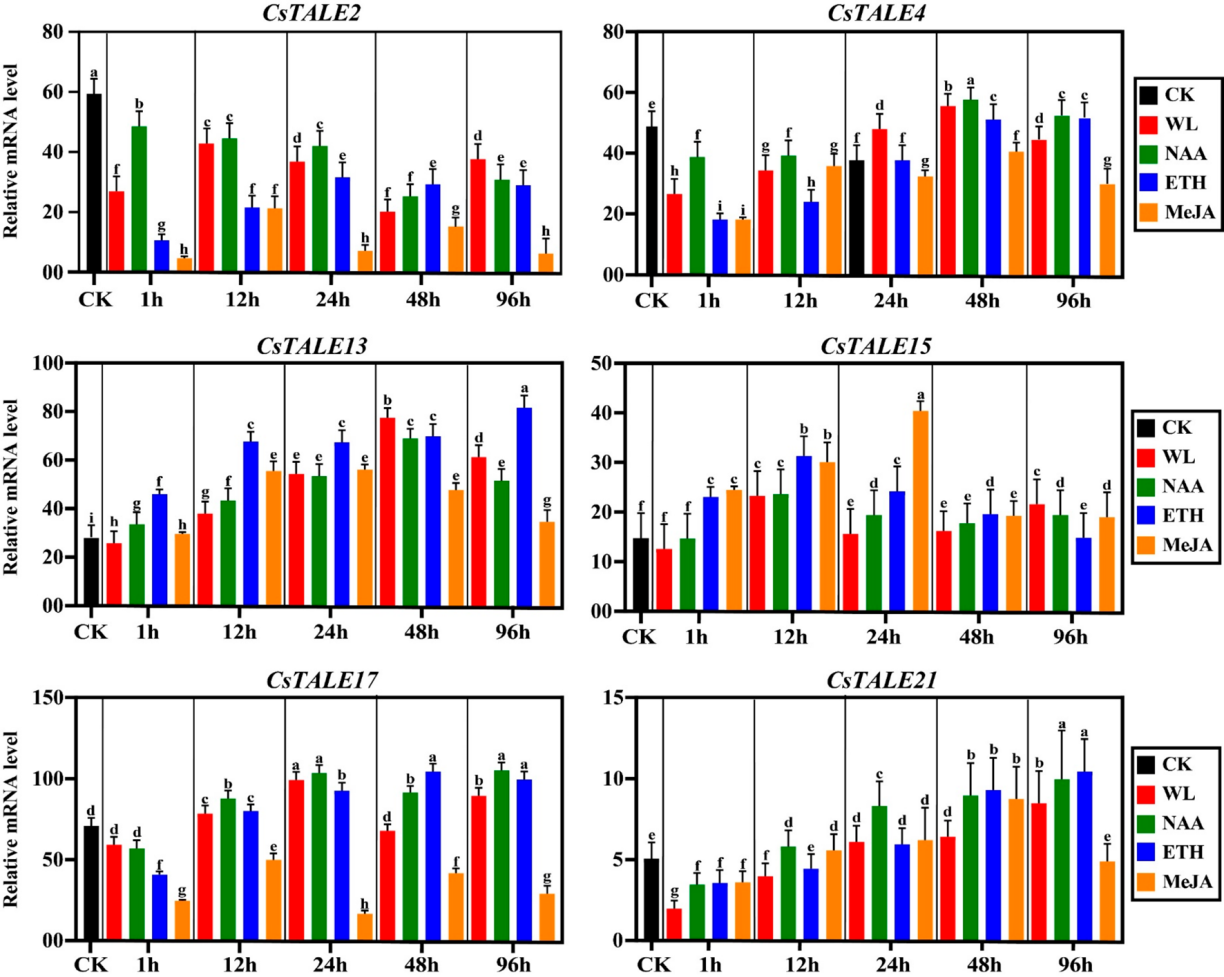
Expression analysis of *CsTALE* genes in response to WL and hormones. The data displays *CsTALE* gene expression under CK, WL, NAA, ETH, and MeJA at 1 h, 12 h, 24 h, 48 h, and 96 h. The histogram bars indicate expression, and the error bars show means ± SEM. The log2 transformation method normalized and converted the RPKM values displayed

The *CsTALE* gene expression under waterlogging in combination with hormones was analyzed. The expression of the *CsTALE1* gene showed lower expression at 1 h after waterlogging (WL), WL-NAA, WL-ETH, and WL-MeJA than the CK (control). At 12 h, the expression was higher under WL and WL-NAA but suppressed again under WL-ETH and WL-MeJA. At 24 h, 48 h, and 96 h, the transcription level of the *CsTALE1* gene was induced under WL, WL-NAA, and WL-ETH compared to WL-MeJA. The *CsTALE4*, in a similar fashion, expressed more in CK than WL, WL-NAA, WL-ETH, and WL-MeJA at 1 h. However, under 12 h, 24 h, 48 h, and 96 h, its mRNA level was sharply induced under WL, NAA, and ETH but reduced under MeJA (Fig. 12). The *CsTALE13* showed similar expressions in CK and WL at 1 h but were stimulated in response to WL-NAA and WL-ETH. The *CsTALE17* displayed the most significant expression in response to WL, WL-NAA, and WL-ETH. The *CsTALE17* was upregulated at all the time points in the WL, WL-NAA, and ETH treated, whereas substantial suppression was observed in the treated cucumber. There was no significant difference in the expression of *CsTALE15* and *CsTALE21* among the treatments.

### *CsTALE* genes expression hormonal applications, NaCl, and silica stress

**Fig. 13:**
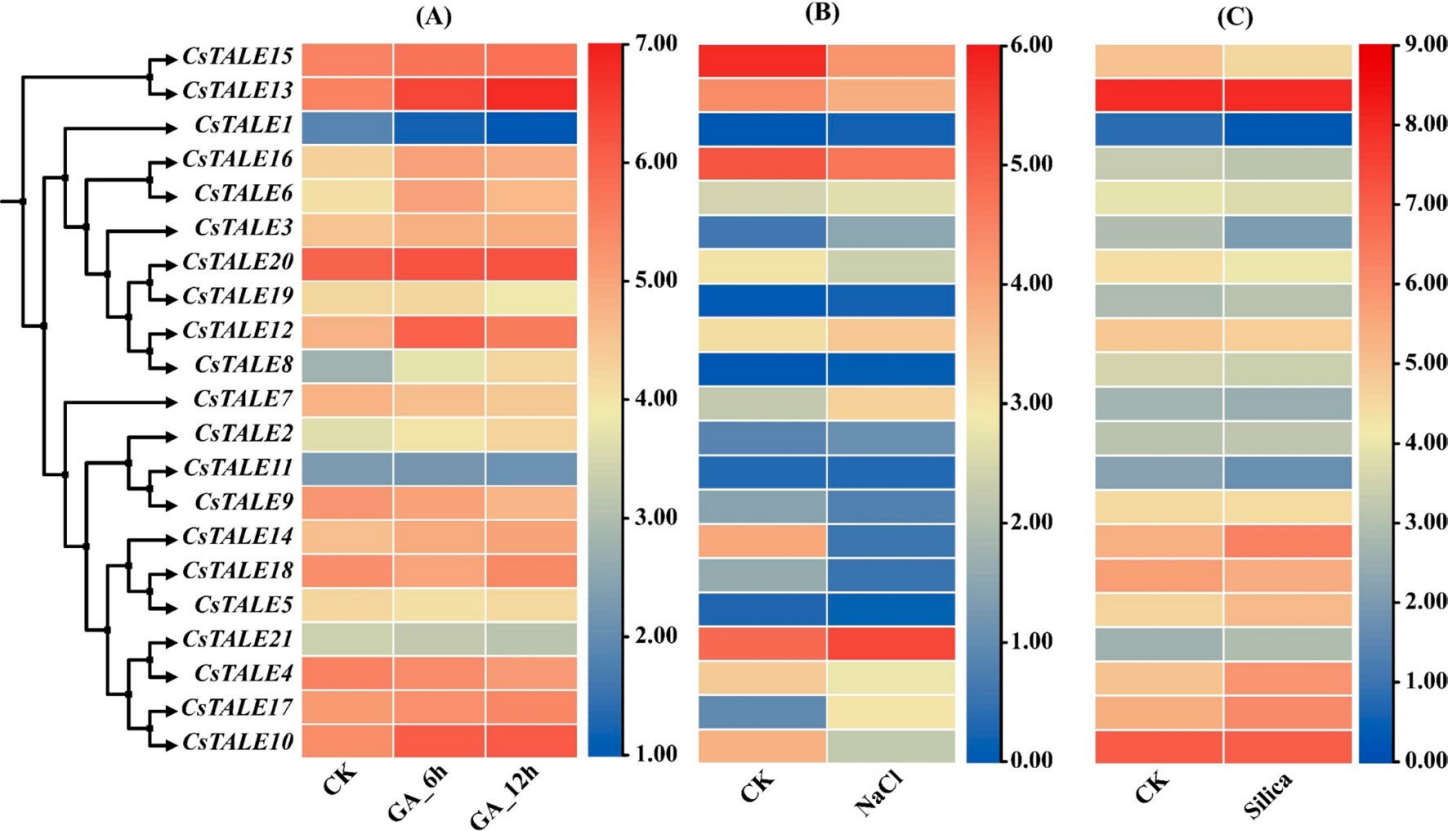
Expression analysis of *CsTALE* genes in response to (**a**) GA treatment, (**b**) NaCl Stress, and (**c**) Silica stress. The expression data was retrieved from the cucumber genome database. The Log^2^ fold normalized data was presented in the form of a heatmap using TBtools-II software (Version 2.027)

To further elucidate the potential role of the *CsTALE* gene family role in regulating the response of *C. sativus* in response to silica stress, NaCl stress, and the GA hormone treatment. We retrieved the expression data from the cucumber genome database and presented it in the form of a heatmap; the heatmap color from blue to red represents the expression percentages (Fig. 12). Under silica stress, only two genes, *CsTALE13* and *CsTALE10*, displayed elevated expression. Several other genes, including *CsTALE4, CsTALE7*, and *CsTALE14*, were observed with moderate expression, whereas the rest of the *CsTALE* family genes were observed with lowest to no expressions (Fig. 13C).

CsTALE gene family members also observed a similar expression trend in response to NaCl stress. In comparison with the control group, fewer genes were highly expressed in response to NaCl stress, such as *CsTALE21* (Fig. 13B). Numerous other genes were also found with moderate expressions, such as *CsTALE13, CsALE15*, and *CsTALE16.* Other members of the *CsTALE* gene family were found to have the lowest or no transcription. Finally, under the GA treatment, the *CsTALE* gene family members showed similar expression trends in both 6 h and 12 h treatments in comparison with the control (Fig. 13A). A single gene, *CsTALE13*, showed the dominant expression in both 6 h and 12 h, followed by *CsALE10, CsTALE12, CsTALE15*, and *CsTALE20* with moderate expression. The *CsTALE11* gene was observed with the lowest expression, and the rest of the *CsTALE* gene family members were found to the moderate to lowest expression, suggesting that the GA-induced *CsTALE* gene family is crucial in regulating the response of *C. sativa* to stressful conditions.

## Discussion

### TALE genes are widely distributed across the genome

Research on TALE, a widely dispersed gene family in eukaryotes, accelerated in the early 20th century [27]. Herein, the isolation of 21 *CsTALE* genes from the cucumber genome database was put forward for comprehensive bioinformatic analysis [28]. As expected, they are all predicted to be located in the nucleus (Table 1) and distributed randomly across 7 chromosomes (Fig. 4).

We built an unrooted phylogenetic tree to investigate the evolutionary relationships of the proteins encoded by the 21 genes. In contrast to earlier research, we categorized these proteins into seven distinct groups [29, 30]. The KNOX genes are categorized into two groups, whereas the BELL genes are further classified into five groups (Fig. 2). The annotations of the SMART database and the ML-phylogenetic tree constructed using the best model also support our classification results [20, 31, 32]. Each of the classes is represented by specific domains or domain combinations.

Gene structure study revealed that every *CsTALE* gene has an intron. Genomic architectures are highly conserved throughout members of the same family. Exon lengths are also quite similar among closely related members of the phylogenetic tree (Fig. 5). Consistent with earlier research on the poplar TALE family, protein motif analysis and annotation showed that members of the same class have identical protein motifs [33].

The *cis*-elements bind transcription factors selectively at the gene promoter region, regulating gene transcription. Several *cis*-elements associated with hormonal response and abiotic stress were discovered in the *CsTALE* promoter sequence, including methyl jasmonate, abscisic acid, and gibberellin (Fig. 8), which is comparable to previous research [11, 34, 35]. Findings suggested a conservative element in the *CsTALE* gene promoter. Research has linked ABRE to ABA induction, drought, and extreme salt stress in plants [36–38]. Several stress-related components are also present, including ARE, MBS, and LTR. According to the findings, the *CsTALE* gene family is involved in the abiotic stress that cucumbers experience. According to gene function prediction and protein-protein network analysis, the *CsTALE* family is key in controlling ovule and inflorescence development. Similarly to the previous study, gene functional prediction and protein-protein network analysis demonstrated that CsTALE15 and CsTALE17 interact with a variety of floral organ proteins [11].

### TALE genes are crucial for growth and development

We investigated the tissue-specific expression of *CsTALE* genes because of the extensive literature demonstrating the TALE gene family’s critical function in plant development and growth. Differences in gene expression patterns among the three organs are clear. Of the 21 *CsTALE* genes, 7 are expressed more in stems than in other tissues (Fig. 11A). To guarantee accurate regulation, TALE expression is closely controlled by upstream genes and other internal and external variables. Many upstream regulators of TALE have been found to have a detrimental effect. The YABBY gene is a negative regulator of the TALE gene that inhibits the expression of class I KNOX genes *(STM, BP/KNAT1*, and *KNAT2).* Mutations in this gene cause improper lateral meristem development and the loss of lateral meristem polarity [39]. AS1 controls *A. thaliana* and Chinese cabbage (*Brassica rapa*) leaf dorsoventral polarity [39]. Multiple KNOX genes (*GhKNL2/3/4*) that are similar to *AtKNAT1* were markedly elevated in GhAS1 or GhAS2 silenced cotton (*Gossypium hirsutum*) [39]. One negative regulator of blooming transition in tobacco (*Nicotiana tabacum*), the *SHORT VEGETATIVE PHASE* (SVP), controls pedicel length by directly inhibiting the *KNAT1* gene NtBPL [39]. The gynoecium developmental program encourages the proper patterning of future fruit once it is established. TALE transcription factors are required for the formation of a number of distinct tissues [40]. At stage 6, the gynoecium creates a ridge of elevated cells around a central cleft and develops replum, valve borders, and valves. In the transverse plane, the adaxial inner side of the replum has a typical meristematic layered structure [40] and accordingly expresses the meristematic genes STM, CLV1/2, and CRN [41]. Only four genes were expressed at a high level in fruit tissue, suggesting their role in fruit development. The study by Jia et al. (2021) advocated the role of *MdTALE* genes in the fruit organ development of apple *(Malus domestica)* [42].

### TALE genes regulate plant response to waterlogging stress

One significant phenotypic response to waterlogging stress is the formation of adventitious root (AR) in cucumber. The formation of AR is greatly influenced by the local production and movement of the plant hormone auxin [21]. Endogenous auxin in the hypocotyl rose 72 h post-waterlogging (WL) stress. The use of 10 mg/L of 1-naphthylacetic acid (NAA) improved AR formation [17]. Further investigation showed that auxin treatment elevated ethylene biosynthesis genes (*CsACS1, CsACS2, CsACO5*) and ROS signaling genes (*CsRBOHB* and *CsRBOHF3*) under WL stress. Treatment with 1-naphthylphthalamic acid (NPA), on the other hand, greatly reduced AR development [20]. Although exogenous NAA enhanced the production of AR, no elongation was seen. As the research showed, sugar treatment has been successful in achieving elongation of the AR [17]. A decrease in AR development and elongation was seen upon shoot removal. By dramatically increasing the expression of *CsPIN1, CsPIN1b, CsPIN8, CsARF5, CsARF6*, and *CsSAUR29*, sugar treatment (300 µL) positively influenced AR formation and elongation [17]. On the other hand, jasmonate, also known as MeJA, is shown to be a negative regulator of WL stress [20]. In our study, the *CsTALE13* and *CsTALE17* were significantly upregulated under WL, NAA, and ethylene (ETH) but not MeJA at all the timepoints (Fig. 12). We speculate that *CsTALE13* and *CsTALE17* could be potential candidates in enhancing cucumber tolerance to WL.

## Conclusion

A total of 21 *CsTALE* genes were identified in the cucumber genome and categorized into 7 groups according to their evolutionary relationship. The presence of hormonal and stress-responsive *cis-*acting elements makes them key regulators of growth and immunity trade-off. The sponging of miR167 and miR319 to the *CsTALE16* and *CsTALE15* advocates the involvement of *CsTALE* genes in posttranscriptional regulation of growth and stress-related processes. The significant upregulation of *CsTALE17* under WL and hormones makes it a possible candidate for functional studies to generate stress-resilient cucumber lines.

## Materials and methods

### Identification of *TALE* genes in *Cucumis sativus*

The entire set of sequence information came from the cucumber genome database (http://cucurbitgenomics.org/organism/2). 19 AtTALE proteins sequences from Arabidopsis were obtained from the Arabidopsis Information Resource (TAIR) website. The *CsTALE* genes in cucumbers were located using a two-stage BLAST search. For the first step, Arabidopsis TALE was utilized in order to look for potential cucumber TALE [43]. Following this, we used BLASTP (e-value, 1e-5) from the National Center for Biotechnology (NCBI; https://www.ncbi.nlm.nih.gov/) to further identify all potential cucumber TALE. Lastly, the SMART (http://smart.embl.de/) and Pfam (http://pfam.xfam.org/) databases were used to corroborate the candidate proteins [44, 45]. Additionally, the physiochemical properties of the TALE proteins in the studied plants were discovered using the ExPASy online server (http://web.expasy.org/protparam/*).*

### Phylogenetic tree, conserved motifs, gene structure, and *cis*-acting elements analysis

Phylogenetic analysis was conducted using TALE protein sequences from the following plants: 21 CsTALE (*C. sativus*), 19 CmTALE (*Cucumis melo*), 23 SlTALE (*Solanum lycopersicum*), and 19 AtTALE (*A. thaliana*). The neighbor-joining (NJ) approach was used to create the phylogenetic tree in MEGAX software v.10.1.8 (https://www.megasoftware.net/). A bootstrap test with 1000 iterations was then performed. Utilizing the Evolview V3 (https://www.evolgenius.info//evolview/#login) display format, the results were prepared for presentation [46, 47]. Using TBtools-II software (Version 2.027), we were able to determine the structure of the cucumber *CsTALE* genes [48]. For the purpose of predicting conserved motifs of the *CsTALE*, the online application MEME, version 5.0.5 (http://meme-suite.org/tools/meme), was utilized. Also, using Gene Structure Display Server 2.0 (GSDS) (http://gsds.cbi.pku.edu.cn), we compared the gene sequences to the predicted coding sequences in order to conduct the gene structure analysis, which included exon and intron analysis [49, 50]. Finally, the PlantCARE database (http://bioinformatics.psb.ugent.be/webtools/plantcare/html/) was utilized to examine the *cis*-acting elements in the 1.5 kb promoter upstream region of *CsTALE* genes with start codon ATG [20].

### Chromosomal localization and prediction of miRNA target genes

Using TBtools-II, we were able to obtain the physical information of the *CsTALE* genes on the chromosomes [48]. A final step involved blasting the CDS sequence of 21 *CsTALE* genes using the online psRNATarget service (http://plantgrn.noble.org/psRNATarget/) in order to identify the cleaved miR319 and miR167 genes. The online server string (https://string-db.org) identified interaction proteins with CsTALE proteins [51].

### Plant material and hormonal + waterlogging treatment

*C. sativus* seeds of the ‘CCMC’ variety (Chinese long) were used for the investigation. All fully developed plants were cultivated in the greenhouses of the Jiangsu Academy of Agriculture Science under natural photoperiod circumstances. For tissue-specific expression analysis, we used forceps to collect mature plants’ roots, stems, leaves, male flowers, and fruits from the field and then placed them in liquid nitrogen. Throughout the experiment, the seedlings were placed in 7 cm diameter pots that contained peat, vermiculite, and perlite in a 3:1:1 ratio (volume/volume). The pots were then placed in growth chambers that were maintained at a constant day temperature of 26 ± 2°C and a night temperature of 18°C, with a relative humidity ranging from 70 to 85%. The 100 µmol/L MeJA, 100 µmol/L NAA, and 100µmol/L ETH (Solarbio, Beijing, China) were used for treatment on the three-week-old seedling stage. For waterlogging stress the *C. sativus* was grown in plastic pots containing a 1:3:1 (v/v/v) mixture of peat, vermiculite and perlite. The pots were placed in a growth chamber with an interior humidity of 80% ± 5%, temperature of 28°C/20°C (14 h light/10 h dark) and light intensity of 300 µmol m^− 2^ sec^− 1^. For waterlogging treatment, the potted seedlings at 21 days after germination (with three true leaves) were moved into plastic cups filled with water to the top of the hypocotyls (approx. 4 cm above the soil surface). The volume of water was kept constant throughout the experiment. Plants were placed in the same cups for control treatments without adding extra water following the same procedure of Xu et al (2023) [52]. All samples were immediately frozen in liquid nitrogen and stored at -80°C until use.

### Quantitative real-time PCR (qRT-PCR)

Using a plant total RNA purification kit from Tiangen (Beijing, China), we followed the manufacturer’s instructions to isolate the total RNA. Gene expression was measured using SYBR® Premix Ex Taq™ II (TaKaRa, China) and an iQ™ 5 Multicolor real-time PCR detection system (Bio-Rad, USA). Using the 2^−ΔΔCT^ technique [53], the relative expression of genes was determined. In keeping with our prior research, we employed three biologically separate replicates for each treatment and normalized the mRNA abundance of each sample using the internal reference gene (Actin) [54].

### Statistical analysis

The SPSS software (version 25.0, SPSS Inc., USA) was used for statistical analysis (ANOVA) and to determine statistical significance (*P* 0.05). Means with standard deviations (SDs) from three biologically independent replicates were used to express the results for all assessed parameters. According to Sharif et al. (2021) [55], the graphical representation was created using GraphPad Prism (version 9.4.1) from GraphPad Software, Inc. in La Jolla, CA, USA.

### Electronic supplementary material

Below is the link to the electronic supplementary material.

Supplementary Material 1

## Data Availability

All data generated or analysed during this study are included in this published article.

## Declarations

Our experiment follows the with relevant institutional, national, and international guidelines and legislation.
