# Supplementary figures and images for: TALE gene family: identification, evolutionary and expression analysis under various exogenous hormones and waterlogging stress in Cucumis sativus L
Source: BMC Plant Biol. 2024 Jun 15;24:564. doi: 10.1186/s12870-024-05274-3 (PMC11179211; doi:10.1186/s12870-024-05274-3)

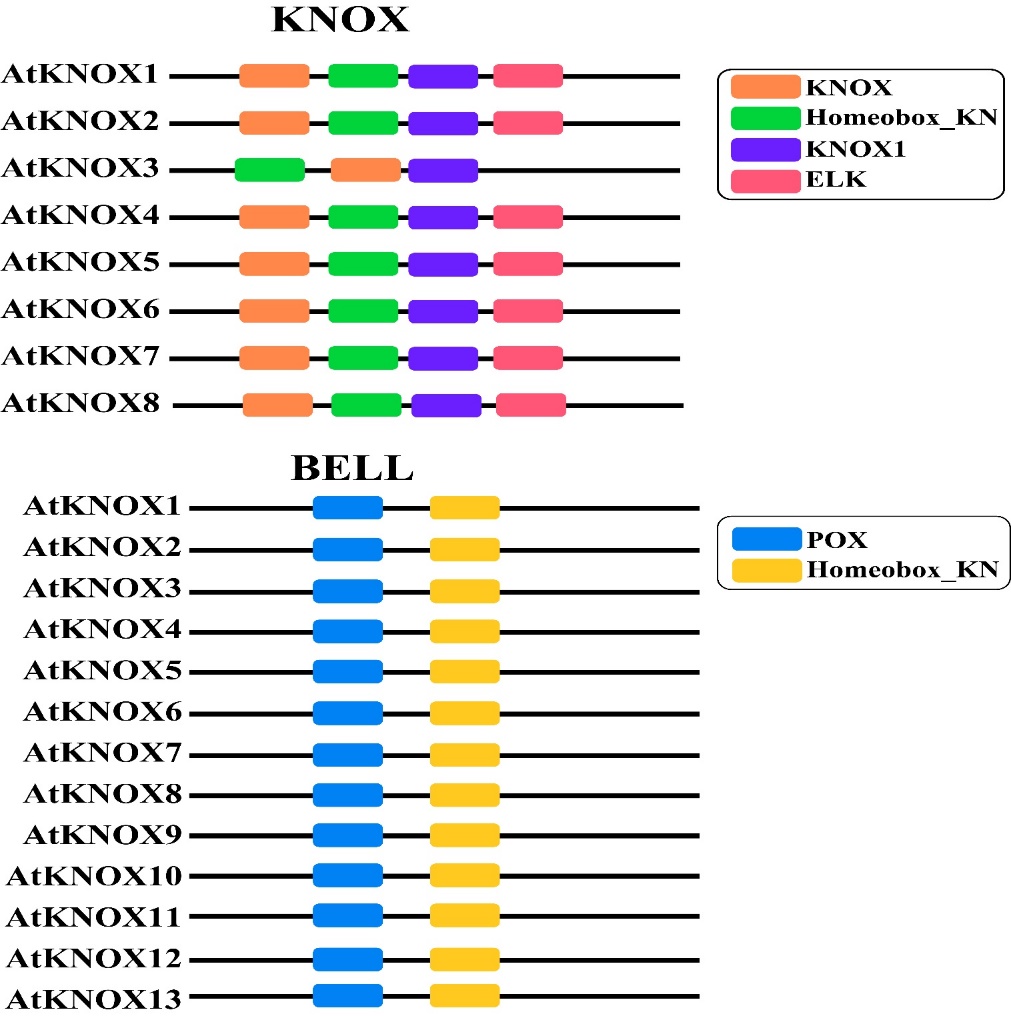


Figure S1: Conserved domains of the AtTALE gene family.

Supplement: Supplementary file 1 — Supplementary Material 1 [file 12870_2024_5274_MOESM1_ESM.docx]
